# CUPRAC-Reactive Advanced Glycation End Products as Prognostic Markers of Human Acute Myocardial Infarction

**DOI:** 10.3390/antiox10030434

**Published:** 2021-03-11

**Authors:** Govigerel Bayarsaikhan, Delger Bayarsaikhan, Pyung Chun Oh, Woong Chol Kang, Bonghee Lee

**Affiliations:** 1 Center for Genomics and Proteomics, Lee Gil Ya Cancer and Diabetes Institute, School of Medicine, Gachon University, Incheon City 406-840, Korea; govigerel.b@n-sage.com (G.B.); delger.b@n-sage.com (D.B.); 2 Gil Medical Center, Department of Cardiology, Gachon University of Medicine and Science, Incheon City 405-760, Korea; likemed@gilhospital.com

**Keywords:** albumin, glycation, CUPRAC, human serum, AMI

## Abstract

Cardiovascular disorders, especially acute coronary syndromes, are among the leading causes of mortality worldwide, and advanced glycation end products (AGEs) are associated with cardiovascular disease and serve as biomarkers for diagnosis and prediction. In this study, we investigated the utility of AGEs as prognostic biomarkers for acute myocardial infarction (AMI). We measured AGEs in serum samples of AMI patients (*N* = 27) using the cupric ion reducing antioxidant capacity (CUPRAC) method on days 0, 2, 14, 30, and 90 after AMI, and the correlation of serum AGE concentration and post-AMI duration was determined using Spearman’s correlation analysis. Compared to total serum protein, the level of CUPRAC reactive AGEs was increased from 0.9 to 2.1 times between 0–90 days after AMI incident. Furthermore, the glycation pattern and Spearman’s correlation analysis revealed four dominant patterns of AGE concentration changes in AMI patients: stable AGE levels (straight line with no peak), continuous increase, single peak pattern, and multimodal pattern (two or more peaks). In conclusion, CUPRAC-reactive AGEs can be developed as a potential prognostic biomarker for AMI through long-term clinical studies.

## 1. Introduction

Acute myocardial infarction (AMI) is a disease with both high rate of mortality and morbidity due to wide range of factors, such as diabetes, obesity, oxidative stress, lifestyle, genetic inheritances, high level of cholesterol and triglyceride in blood, etc. [1,2]. The pathogenesis of AMI remains unclear, but nevertheless the involvement of post translational modification of proteins, such as glycation/glycoxidation, have been known and resulted that level of glycated albumin in human serum is associated with outcomes and deaths of the cardiovascular disorders [3]. Furthermore, with the advances in molecular biology and medicine, a large and attractive body of evidence for using cardiac injury biomarkers for prognosis of AMI have been accumulated and, among them, well known biomarkers are aspartate transaminase, creatine kinase, lactate dehydrogenase, creatine kinase MB (CK-MB), and cardiac troponins [4]. In addition, it is known that an elevated level of inflammatory biomarkers (i.e., IL6, and IL8) is followed by AMI incidences [5]. Recent findings suggest that silencing the receptor for advanced glycation end products (RAGE) may discourage inflammation induced by advanced glycation end-products (AGEs). Moreover, significant reductions were observed in infarction size after ischemic reperfusion injury in RAGE knockout mice, compared to wild-type mice [6,7]. Besides, it was found that AGEs generated after myocardial infarction are involved in cellular changes, leading to functional loss, cardiomyocyte apoptosis, and negative remodeling of the heart in subsequent days [8,9]. Unfortunately, there is almost no simple diagnostic strategy to manage infarctions by controlling AGE concentrations, despite the continued and rapid increase in annual AMI incidence.

The AGEs are formed by non-enzymatic reactions between carbonyl groups of reducing sugars and nucleophilic amino groups of proteins, lipids or nucleic acids in the Maillard reaction, in which browning, fluorescence and cross-linking are characteristic [10]. Many different AGEs are generated through enolization, dehydration, cyclization, fragmentation and/or oxidation. Reversible aldimine or Schiff’s base intermediates formed in the early stages of the Maillard reaction are converted by intramolecular rearrangements to more stable Amadori products, which in turn undergo further dehydration and oxidation to form highly stable AGEs. This is why the acronym AGEs is also commonly understood in the literature as advanced glycoxidation end-products. Highly reactive dicarbonyl compounds capable of rapidly forming AGEs include glyoxal (GO), ketoaldehydes like methylglyoxal (MG) and deoxyosones like 3-deoxyglucosone (3-DG). While 3-DG is formed by the non-oxidative rearrangement and hydrolysis of Amadori products, MG can be produced both by the autoxidation of carbohydrates and by lipid peroxidation. 3-DG and MG react with lysyl amino groups of proteins to form Nε-(carboxymethyl)lysine (CML) and Nε-(carboxyethyl)lysine (CEL), respectively [11,12]. CML was found as the most abundant AGE in human plasma [13]. Because CML and CEL may derive from free-radical attack to derivatives of both carbohydrate and lipid metabolism, they are referred to as mixed AGEs and/or advanced lipoxidation end products (ALEs) [14]. Moreover, AGEs accumulate in tissue proteins with slow turnover rates (i.e., albumin and collagen), and it is reported that pathogenesis of various disorders, including atherosclerosis and microvascular complications, are initiated or enhanced due to the mutual effect of glycation with other toxic reactions such as oxidation and lipoxidation [15]. On the other hand, because of their diversity and functional activities, there is no standardized potential method for measuring AGEs. Thus, instrumental analysis (i.e., mass spectrometry or associated combined techniques with matrix-assisted laser desorption/ionization time-off light device, traditional and high-performance liquid chromatography, and higher energy collisional dissociation), electrochemical sensors, and molecular biological methods, such as immunoblotting assays, are among the most accessible biomedical techniques [16,17,18,19,20]. These methods can determine AGEs with high accuracy and precision; however, they have several disadvantages, such as time and cost consumption, requirement of highly developed instruments, well-equipped laboratories, specialized experts, and their results are difficult to interpret.

Therefore, in this study, we introduced the cupric ion reducing antioxidant capacity (CUPRAC) assay as a simple, rapid, and cost-effective method for measuring AGEs in BSA and human serum samples of AMI patients. The CUPRAC method is a globally used optical method and its principle is based on measuring the absorbance of the Cu (I)-Nc complex generated by the reduction reaction of Cu (II)-Nc, using a wavelength of 450 nm [21]. This method was developed by a research group led by Professor Resat Apak and has been successfully applied to measure the antioxidant activity of bioactive compounds in bio-logical samples or foods, while showing its potency for determining oxidative damage in DNA and proteins [22,23,24]. Furthermore, this system has remarkable merits; for example, the assay works considerably well at physiological pH (approximately 7.0), it uses stable reagents, and because of its low redox potential, CUPRAC reacts with a wide range of compounds, including thiols, mono- and polyphenols, carbonyls, and imidazole ring-containing compounds [25,26]. With respect to their functional groups, certain analogs have been described as AGE compounds and have been recognized for their contribution to the pathogenesis of AGE-associated disorders. For example, hydroimidazolones (i.e., glyoxal-, methylglyoxal-, and 3-deoxyglucosone-derived hydroimidazolones), bis-lysyl imidazolium cross-links (i.e., non-fluorescent methylglyoxal-derived lysine dimer, glyoxal-derived lysine dimer, and 3-deoxyglucosone-derived lysine dimer), and fluorophores, such as argpyrimidine and pentosidine [27,28,29]. Thus, based on the similarities in structures of CUPRAC-tested antioxidants and certain forms of AGEs, we proposed prospective application of the CUPRAC assay for detecting glycation levels of proteins. We expect that our findings will contribute to the development of CUPRAC-based AGE assays in the near future to predict the outcomes of cardiovascular remodeling and support physicians in the timely selection of appropriate therapeutic strategies.

## 2. Materials and Methods

### 2.1. Reagents

All chemicals used were of analytical grade. Chemical reagents, including glucose (C_6_H_12_O_6_), sucrose (C_12_H_2_O_11_), sodium dihydrogen phosphate (NaH_2_PO4 × 2H_2_O), disodium hydrogen phosphate (Na_2_HPO_4_ × 22H_2_O), sodium azide (NaN_3_), copper chloride dihydrate (CuCl_2_ × 2H_2_O), neocuproine (Nc) hydrochloride monohydrate (C_14_H_12_N_2_ × HCl × H_2_O), ascorbic acid (AA) (C_6_H_8_O_6_), and trichloroacetic acid (C_2_HCl_3_O_2_) were obtained from Sigma Aldrich (St. Louis, MO, USA). A 1M Tris-HCl buffer (pH = 7.0) and bovine serum albumin (BSA; Fraction V) were purchased from Biosesang (Gyeonggi, Republic of Korea) and MP Biomedical (Irvine, CA, USA). The commercial standard for AGE-BSA (ab51995) was obtained from Abcam (Cambridge, UK). The Pierce BCA Protein Assay Kit (cat. no. 23215) was obtained from Thermo Fisher Scientific (Waltham, MA, USA).

### 2.2. Albumin Glycation In Vitro

AGE albumin was produced according to a previously published method with minor modifications [30]. Briefly, the compound was produced by incubating 2–10 mg/mL BSA in 0.2% sodium azide containing 0.1 M phosphate buffer (pH = 7.4) for 50 days in the presence of glucose (Glu) (0.1 M) at 37 °C in the dark. In the experiments designed to de-termine AA accelerating activity on protein glycation, 0.05 M of a stock solution for AA was prepared. After this, 50, 100, 250, and 500 µL aliquots of the stock were added to the reaction mixtures. The final volume of the mixture was 5.0 mL.

### 2.3. Human Serum Sampling and Preparation

Human serum samples were collected from 27 patients hospitalized due to AMI at the Gil Hospital, Incheon City, Republic of Korea. The study was approved by the Internal Review Board of Gil Hospital (no.: GDIRB2018-406). Blood sampling was performed on the day of the AMI incident (d 0), and two days, two weeks, one month, and three months after that. Serum was separated from plasma using high-speed centrifugation and was stored at −80 °C until analysis. The patients were hospitalized for six days, during which time their troponin I, creatine kinase-MB (CKMB) levels, and the severity of coronary artery disease (CAD) were monitored. According to the manufacturer’s guidelines, protein concentrations of serum samples were determined using a BCA kit (cat. no. 23215, Thermo Fisher). The serum samples were then diluted to a final concentration of 2 mg/mL of protein using autoclaved distilled water to a final volume of 100 µL, which was placed on ice. CUPRAC was measured directly using the serum dilution method; all experiments were repeated three times, and the average values were used for data analysis.

### 2.4. CUPRAC Measurement of AGEs in Protein Samples

**Figure 1:**
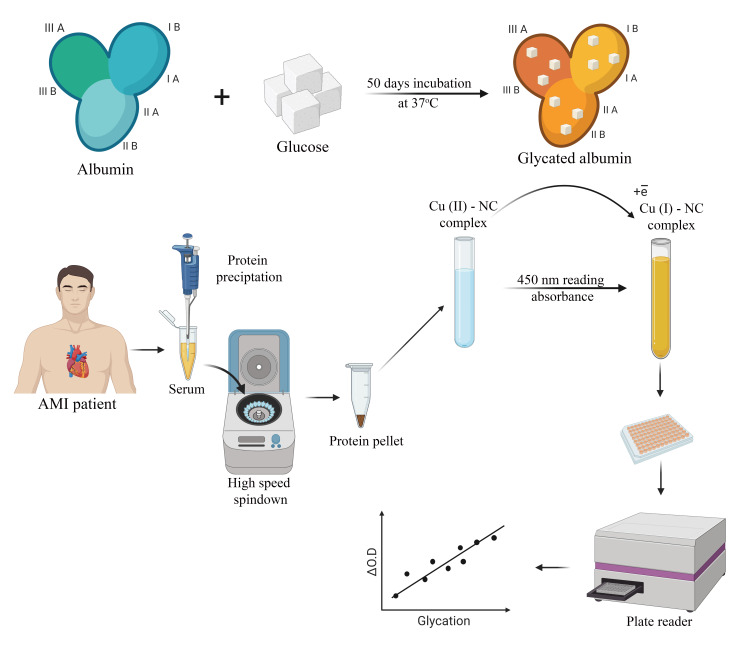
Experimental procedure for determining advanced glycation end products (AGEs) using a cupric ion reducing antioxidant capacity (CUPRAC) assay; visualized using Biorender software [31].

The CUPRAC assay was performed as previously described [24], with minor modifications. Briefly, 100 µL sample was withdrawn from the incubated mixtures, and protein content was precipitated using 40 µL 0.6M trichloroacetic acid solution to determine AGEs formed on BSA. The supernatant of the solution was then completely separated from the precipitated protein using a micropipette after centrifugation at 12,000 rpm for 1.5 min. After this, the protein pellet was dissolved and was vortexed in 350 µL 1M Tris HCl buffer (pH = 7.0). When the pellet was completely dissolved, 125 µL 10 mM copper sulfate and 125 µL Nc (dissolved in a 1:20 mixture of ethanol and distilled water) was added to the protein tube, and the reaction was incubated at 50 °C for 45 min. The optical density of the solution was measured at 450 nm using an enzyme-linked immunosorbent assay (ELISA) microplate reader. All measurements were performed on BSA samples prepared in parallel tubes, and average values were used for data analysis. The experimental procedure is visualized in Figure 1.

### 2.5. Statistical Analyses

Descriptive analysis was performed using Microsoft Excel software. Spearman’s correlation coefficient was calculated using the following equation to define the pattern type of AGEs formed in patients after AMI.

(1) $$r_{s}=\;1-\frac{6\sum_{i}d_{i}^{2}}{n\left(n^{2}-1\right)}$$

where *r_s_* is the Spearman’s rank correlation coefficient, *d_i_* is the difference between the two series, and n represents the number of sampling days. Coefficients in the ranges 0–0.39 and 0.40–0.59 were considered to indicate weak and moderate correlations, respectively, and those in the ranges 0.6–0.78 and 0.8–1.0 were considered to indicate strong to very strong correlations, respectively [32].

## 3. Results and Discussion

### 3.1. Optimization of CUPRAC Assays for Detecting AGEs

**Figure 2:**
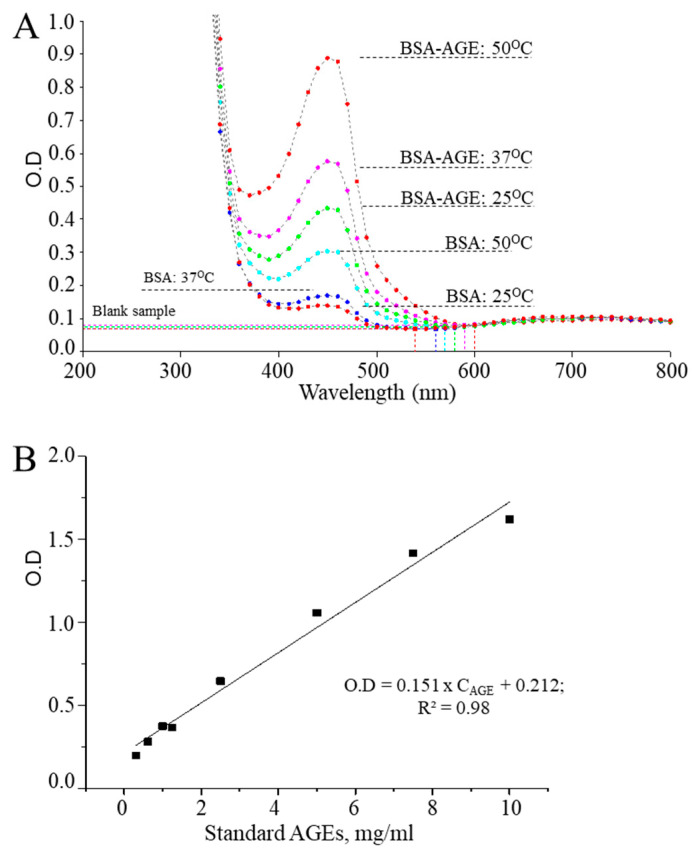
Applicability of the CUPRAC assay on glycated albumin; (**A**) spectral scanning of bovine serum albumin (BSA) and AGEs followed by different incubation temperatures of CUPRAC system (2 mg/mL BSA + 0.5 M glucose; glycation after 14 days); (**B**) calibration curve for CUPRAC measurement of the standard BSA-AGEs.

The CUPRAC assay was successfully applied to measure antioxidant activity of foods and bioactive compounds, while also showing its potency for determining oxidative damage of DNA and proteins using various techniques such as electrochemical or optical methods [21,22,23,24]. In the latter case, CUPRAC-reactive products generated by protein oxidation (in short time interval) were not detected in the precipitated form of albumin, but were present in the supernatant. In contrast, in the current study, we found that CUPRAC-reactive AGEs generated by albumin and glucose reactions can be measured in the precipitated form of the protein. Depending on the sample type and purpose of the analysis, the method is typically used with incubation at room temperature, i.e., 37 °C or 50 °C [33,34]. In the present study, we used incubation at 50 °C due to the highest optical density values at this temperature. The spectral scanning of the CUPRAC system for glycated albumins showed maximum absorbance at similar wavelengths (450 nm) using an ELISA plate reader and the CUPRAC assay in the spectrophotometric analysis (Figure 2A). Un-der CUPRAC assay-optimized conditions, methodological validation was performed using an AGE-BSA standard (ab51995; Abcam), which showed a very strong correlation of the concentration of AGE-BSA and the corresponding CUPRAC values (Figure 2B). Com-pared with spectrophotometry, ELISA is cost-effective as it requires only 200 µL (final volume) of the sample for a single reading and is more suitable for daily biomedical application of the CUPRAC method, as it allows simultaneous analysis of up to 96 samples.

### 3.2. CUPRAC Measurement Results of AGEs

**Figure 3:**
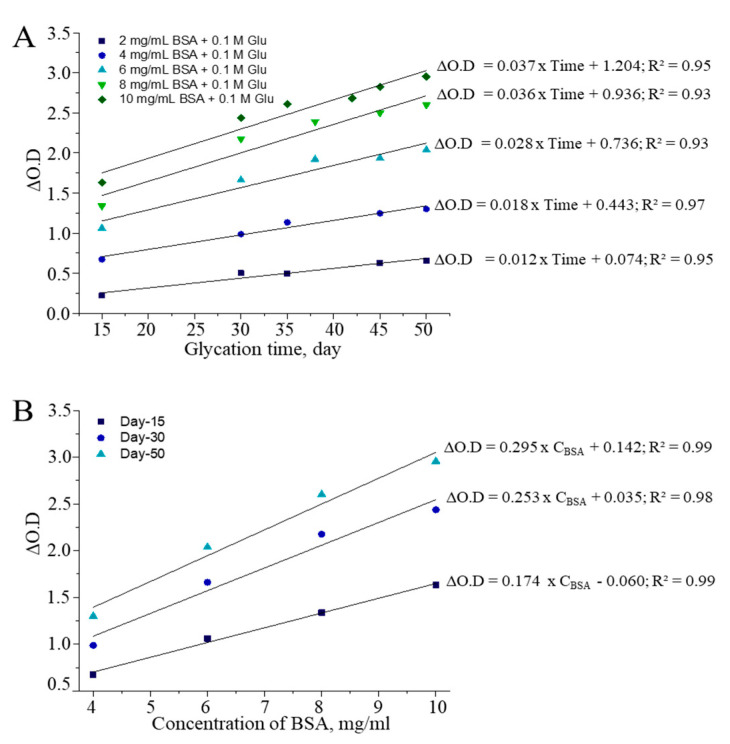
BSA-dependent formation of AGEs (**A**) regarding glycation time; (**B**) within-day correlation of formed AGEs.

Albumin is found in both intra-and extracellular proteins, and its serum concentration is typically within the range of 35–50 g/L in healthy individuals. Furthermore, changes in serum albumin levels are associated with various disorders; for example, albumin concentration increases can be associated with prediabetes development, incident hemodialysis, and administration of certain types of drugs, while a decrease may be observed in patients with acute heart failure, sepsis, and hypoalbuminemia [35,36,37,38,39]. There-fore, we first determined the sensitivity of the CUPRAC assay regarding the glycation re-action that occurred in tubes containing different concentrations of BSA (2–10 mg/mL) and the same amount of glucose (0.1 M) in a phosphate buffer environment (pH = 7.4). A regression analysis revealed that increases in the BSA component of the non-enzymatic glycation system generated significantly higher levels of AGEs than those observed at low albumin levels. Accordingly, when the BSA concentration increased from 2 mg/mL to 10 mg/mL at a consistent glucose concentration, the CUPRAC slope of the regression equation increased from 0.012 to 0.037 (from *R^2^* = 0.93 to 0.97) with respect to the duration of the glycation reaction under the given conditions (Figure 3A). Simultaneously with glycation time, there was strong linearity (from *R^2^* = 0.98 to 0.99) between albumin concentration and generation of CUPRAC-reactive AGEs, according to horizontal or within-day measurements recorded on days 15, 30, and 50 (Figure 3B).

**Figure 4:**
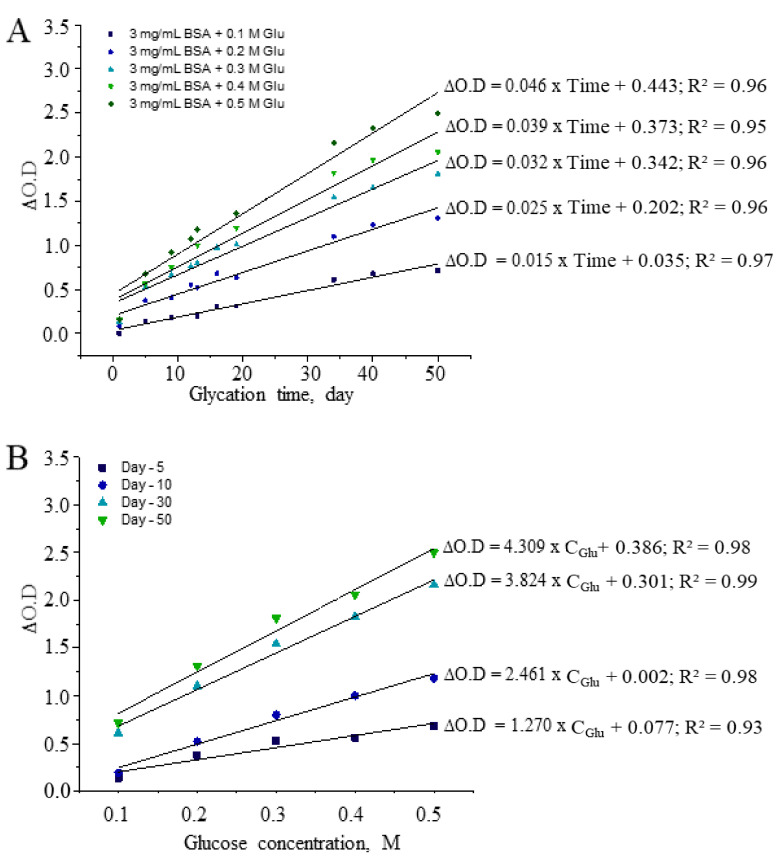
Glucose-dependent glycation of 3 mg/mL BSA (**A**) regarding glycation time and (**B**) within-day correlation of formed AGEs.

This process continues through different routes, depending on the location of glycation. For example, during extracellular glycation, the primary route to AGE formation is Schiff’s base reaction, which further leads to Amadori products with rearrangements. The reaction is mostly linked to high levels of sugar and glucose in the blood. In contrast, in the intracellular environment, AGE formation from glycation is driven by glucose metabolites such as glyoxal, methylglyoxal, and 3-deoxyglucosone [40]. Next, we investigated the sensitivity of CUPRAC as a means of measuring AGEs formed from serum albumin at the fixed concentration of 3 mg/mL in the presence of glucose concentrations varying from 0.1 to 0.5 M. The CUPRAC slope of regression equation ranged from 0.015 to 0.046 (from *R^2^* = 0.95 to 0.97) concerning glycation time and was similar to that observed in the BSA-dependent glycation reaction (Figure 4A). This shows the equal contribution of albumin and glucose to the generation of AGEs, along with glycation time. By contrast, horizontal measurements conducted on glycation tubes containing different amounts of glucose showed strong linearity (*R^2^* = 0.93 to 0.99) (Figure 4B). The slope coefficients of CUPRAC-active AGEs increased substantially with increasing glucose concentration, showing that AGEs are generated depending on the amount of sugar molecules present in vitro.

The CUPRAC system reacted with standard (commercial) AGEs with strong correlation and acceptable regression linearity (Figure 2B); thus, it is possible to estimate the amount of generated AGEs from the regression equation derived from the calibration curve. In this study, the degree of BSA glycation was expressed as the ratio of AGEs to BSA, and the respective results are shown in Table 1.

The CUPRAC assay demonstrated that, compared with the initial amount of albumin, AGE concentrations increased by 1.11-fold to 5.04-fold within 50 days, with respect to increases in glucose molecules. These increases can be associated with a large number of glycation sites of albumin, which, simultaneously, shows the remarkable stability of AGEs [18,41,42]. Currently, approximately 20 types of AGEs have been recognized in human tissues, blood, and foods. Among these, well-known products are CML, CEL, pyrraline, pentosidine, imidazolone, 3-deoxyglucosone-hydroimidazolone-1, glyoxal hydroimidazolone-1, methylglyoxal hydroimidazolone-1, methylglyoxal-derived lysine dimer, argpyrimidine, and pentosidine [43]. High diversity and complexity regarding the formation of AGEs makes it difficult to distinguish them in terms of their CUPRAC reactivity. However, the CUPRAC method is based on an electron transfer mechanism, and on this basis, we expect that only redox-active AGEs are measured by this assay via either distinctive or synergistic mechanisms [44]. Thus, further in-depth studies to examine the specificity of the CUPRAC assay against AGEs will advance biomarker science due to the advantages of this assay, such as rapid response to AGEs, simplicity, and cost-effectiveness. Moreover, it can be applied not only for measuring AGE biomarkers but possibly also for drug discovery regarding diseases associated with AGEs. For example, the CUPRAC method has also been shown to simultaneously measure oxidative protein damage and inhibit antioxidant compound activity [24]. Therefore, it can be applied further to test the effects of bioactive compounds against toxic AGEs.

### 3.3. Ascorbic Acid (AA)-Induced Alterations in AGEs

**Figure 5:**
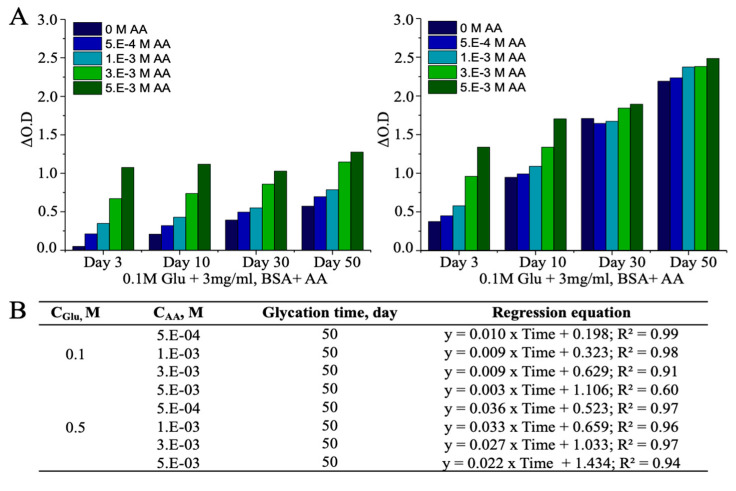
Accelerated formation of AGE albumin in presence of AA; (**A**) AA-induced alteration in AGEs in high and low glucose environments; (**B**) regression equation of CUPRAC measurements of AGEs in the AA-associated glycation system.

Various substances have been identified for their synergetic and deleterious effects on the post-translational modification of albumin by reducing sugar molecules. Among them is AA with its inhibitory effects in vivo and cooperative effects in vitro on the albumin glycation reaction [45,46]. Accordingly, to investigate the effects of external factors on non-enzymatic glycation of albumin using the CUPRAC assay, various amounts of AA were introduced into the reaction mixture comprised of protein (3 mg/mL BSA) and glucose molecules, which after sterilization was incubated in the dark for 50 days in the presence of 0.1 M and 0.5 M glucose. The results obtained with the CUPRAC assay are consistent with previous findings. For example, the amount of CUPRAC-reactive AGEs was significantly increased due to the presence of AA, compared to that in the absence of AA (Figure 5A). In addition, the CUPRAC assay efficiently responded to BSA-AGEs formed in the presence of AA, while it showed high correlation for generated AGEs concerning the concentration of treated AA (5.E-04 to 3.E-03 M) in both high and low glucose environments (Figure 5B). This demonstrates that AA molecules are degraded in physiological pH into their unstable and reactive products, such as dehydroascorbate, 2,3-L-diketogulonate, L-erythrulose, and oxalate, which can initiate further reactions with nucleophilic residues of proteins and accelerate non-enzymatic glycation reactions in vitro [47,48]. In the current study, non-enzymatic glycation reactions proceeded in an exponential intensification mode in the presence of high AA concentrations. The weakest correlation with respect to glycation time was observed for the reaction tube containing the lowest level of glucose (0.1 M) and the highest concentration of AA (5.E-03 M). This suggests that AA may refuel the non-enzymatic glycation of serum proteins by refurnishing the pool of reducing glucose.

In addition, BSA was incubated with the same amount of AAs, but compared to the synergetic effect of AA and glucose, induced glycation to BSA was negligible or relatively low according to the CUPRAC assay (Table 2).

### 3.4. Detection of AMI-Induced AGEs in Human Serum

AGEs have been extensively used as biomarkers for AGE-associated disorders such as diabetes mellitus, inflammation, and neurodegenerative diseases [49,50,51], and successful biomarker diagnosis plays a significant role regarding treatment decisions. AMI patients are at risk of AGE accumulation, and subsequent cell death is an essential factor in the development of their affliction [52]. Recent studies demonstrated that AGEs bind to their cellular receptors to produce advanced glycation end products (RAGE), and the development of AGE-RAGE binding leads to oxidative stress, cell death, and fibrosis [53]. Accordingly, to demonstrate the clinical translation of the CUPRAC assay, AGE components were measured in serum samples of AMI patients using a modified CUPRAC assay as described in the current study. The BSA molecule demonstrated background noise in the CUPRAC system (Figure 2A). Thus, the protein content of human serum samples was determined using a commercial BCA protein assay kit, and all samples were diluted approximately 40-fold to reach a final concentration of 2 mg/mL for analysis of CUPRAC re-active AGEs in the AMI patient’s serum sample. Then, the values of the CUPRAC-reactive AGEs were obtained using the median value of triplicate measurements. Using the CUPRAC system, the average value of optical density increased from 0.56 ± 0.06 on day 0 to 0.66 ± 0.05 on day 90 among the study subjects. The AGE components of human serum samples were calculated according to the calibration equation shown in Figure 2B, and the AGE/BSA ratios are shown in Table 3. The ratio of AGEs to serum total protein (2 mg/mL) was used as a parameter for identifying the alterations in AGE level in human serum and the ratio demonstrated that the level of CUPRAC reactive BSA-AGE was in-creased from 0.9 to 2.1 times between 0–90 days after the AMI incident. Based on the estimated relative standard deviation values, patients were assigned to three groups with respect to their AMI-induced alterations in serum AGE level, i.e., substantially increased (group 1), moderately changed (group 2), and relatively stable (group 3) (Table 3).

**Figure 6:**
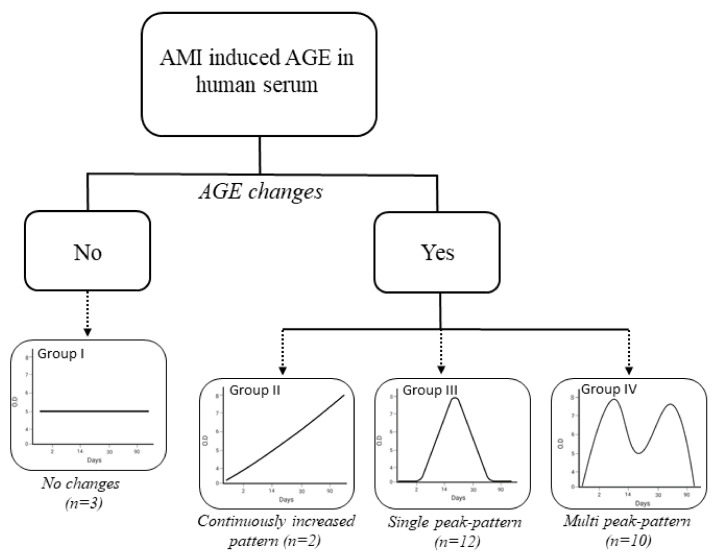
Dominant pattern for generation of AGEs after AMI in human serum.

We performed Spearman’s correlation analysis of AGE concentrations and the time at which AGE concentrations peaked. The most frequently observed changes occurred patients 3, 7, 20, and 28 (Supplementary Table S1), and the results were consistent with the classification shown in Table 3. In summary, based on the glycation pattern and Spear-man’s correlation coefficients, the post-AMI changes in AGE levels were categorized in four main patterns: (i) stable AGE levels (straight line with no peak), (ii) continuous in-crease, (iii) single peak pattern, and (iv) multimodal pattern (two or more peaks), as shown in Figure 6 (Supplementary Figures S1–S4).

In a study by Mulder et al., autofluorescence of the skin was found to increase during post-AMI, and the elevated level of autofluorescence is due to accumulation of AMI-induced AGEs in the skin. The patients were followed up for approximately 400 days, and the findings revealed that probability of survival among individuals with AMI was negatively correlated with dermal autofluorescence values [54]. Similarly, to under-stand the clinical translation of alterations in CUPRAC-reactive AGEs followed by AMI, we compared changes in the levels of other biomarkers and major risk factors of AMI, such as the base and peak values of troponin I and creatine kinase-MB (CKMB), as well as the extent of CAD, with respect to the four patterns described in this study (Table 4). However, the background value of these biomarkers can vary individually, but the peak values play a crucial role for the prognosis [55,56]. Among the four dominant patterns of AGEs (Figure 6), the troponin I level remarkably increased by 107-fold in group III, where-as this increase was estimated approximately 10-fold in the other three groups. By contrast, compared to its base value, the peak value of CKMB increased approximately 70-fold in group IV, and it increased 3- to 10-fold in the other three groups. Among the other forms of CAD, triple-vessel disease represents the highest risk of mortality after AMI [54]. In this study, the number of patients with three vessel diseases was estimated at 33.3–40% in groups III and IV, whereas there were no subjects with three vessel diseases in groups I and II. The results suggest that the pattern of AGEs may be associated the severity of AMI and may also depend on the type of affliction, for instance, whether it is troponin-positive, CKMB-negative (group III, single peak pattern), or a troponin-negative, CKMB-positive (group IV, multi-peak pattern) acute myocardial infarction [57,58,59]. Accordingly, it is suggested that the level of CUPRAC-reactive AGEs remained constant or slightly increased (Supplementary Figures S1 and S2) after the non-critical form of AMI and the severe form of AMI is linked to single- or multi-peak alterations (Supplementary Figures S3 and S4) of AGEs. Taken together, our findings expand the insights produced by previous studies on mortality risk of AMI patients and association with the accumulation degree of AGEs in long-lived proteins (i.e., albumin and collagen) by investigating the correspondence between cardiac prognostic biomarkers and alteration patterns of AGE levels in serum of AMI patients over three months. Nevertheless, it should be noted that the increases in CUPRAC reactive end products of serum proteins measured in this study probably reflect not only increased rates of glycation/glycoxidation, but of secondary factors, since AGEs can arise in vivo during various conditions, including oxidative stress and lipid peroxidation. Consequently, further long-term follow-up studies should be performed to confirm these results, as short-term signal-based prognostic tools have certain benefits over long-term prognostic tools in terms of effective prevention measures.

The increase in AGE concentrations can be associated with frequent and high-dose consumption of accelerator molecules for generating AGEs for in vivo via reactions of proteins with sugar molecules. Our analysis of exposing the system containing a glycated protein to a high amount of AA revealed a considerable increase in AGE concentrations in vitro (Figure 5 and Table 2). In contrast, previous studies showed that continuous de-creases in albumin levels are followed by post-AMI [60]. As shown in this study, decreases in albumin levels were positively correlated with reduced generation of AGEs, as well.

## 4. Conclusions

Our results suggest four possible patterns of changes in serum AGE levels three months after AMI incidents. Using AGE as a biomarker for predicting the prognosis and assessment tool for risk evaluation of patients will bring promising benefits for reducing the morbidity rate of acute coronary syndrome, one of the leading causes of death world-wide. In conclusion, CUPRAC-reactive AGEs can be developed as a potential biomarker for predicting AMI prognosis in long-term clinical studies. A further novelty of this study is the modification of the CUPRAC method for measuring AGEs using an ELISA reader, which allows its use as a simple, rapid, and cost-effective method.

## Figures and Tables

**Table 1:** Estimated amount AGEs and their ratio to BSA in experiments conducted within different ranges of exposure to glucose.

Reaction Mixture	CUPRAC Reactive AGEs, mg/mL				AGEs/BSA			
	D-5	D-15	D-30	D-50	D-5	D-15	D-30	D-50
3 mg/mL BSA + 0.1M Glu	0.48	0.13	2.64	3.33	0.16	0.04	0.88	1.11
3 mg/mL BSA + 0.2M Glu	1.08	2.06	5.87	7.28	0.36	0.69	1.96	2.43
3 mg/mL BSA + 0.3M Glu	2.10	3.89	8.80	10.59	0.70	1.30	2.93	3.53
3 mg/mL BSA + 0.4M Glu	2.32	5.23	10.66	12.20	0.77	1.74	3.55	4.07
3 mg/mL BSA + 0.5M Glu	3.10	6.43	12.89	15.12	1.03	2.14	4.30	5.04

**Table 2:** Estimated amount AGEs and their ratio to BSA in experiments conducted at different glucose concentrations.

Reaction Mixture	CUPRAC-Reactive AGEs, mg/mL			
	Day-3	Day-10	Day-30	Day-50
BSA+ 0.5M Glu + 2.5E-04M AA	1.07	4.86	9.90	13.08
BSA+ 0.5M Glu + 5.0E-04M AA	1.57	5.15	9.48	13.37
BSA+ 0.5M Glu + 1.0E-03M AA	2.42	5.80	9.65	14.29
BSA+ 0.5M Glu + 2.5E-03M AA	4.95	7.44	10.79	14.33
BSA+ 0.5M Glu + 5.0E-03M AA	7.44	9.87	11.12	15.02
BSA+ 2.5E-04M AA	N.A	N.A	N.A	N.A
BSA+ 5.0E-04M AA	N.A	N.A	N.A	0.35
BSA+ 1.0E-03M AA	N/A	N.A	N.A	1.20
BSA+ 2.5E-03M AA	1.73	1.59	0.88	1.51
BSA+ 5.0E-03M AA	4.19	4.52	3.77	2.23

**Table 3:** Ratios of CUPRAC reactive AGEs to total serum protein in human serum; RSD: relative standard deviation.

AGE/total Serum Protein						
**Patient No.**	**Group I (10% ≤ RSD)**					
	**Day 0**	**Day 2**	**Day 14**	**Day 30**	**Day 90**	**RSD, %**
1	1.1	1.6	1.6	1.9	1.7	12.7
3	1.1	1.4	1.5	1.4	1.7	10.3
4	1.1	1.6	1.7	1.8	1.7	12.3
5	1.0	1.5	0.0	1.7	1.3	13.2
13	1.2	1.6	1.3	1.0	1.4	11.1
16	0.9	0.9	1.0	1.0	2.1	26.3
17	1.0	1.1	1.5	1.6	1.4	11.9
22	1.0	1.2	1.3	1.4	1.6	12.1
						
**Patient No.**	**Group II (5% < RSD < 10%)**					
	**Day 0**	**Day 2**	**Day 14**	**Day 30**	**Day 90**	**RSD, %**
6	1.3	1.6	1.6	1.5	1.6	5.4
9	1.2	1.6	1.5	1.5	1.7	9.2
10	1.5	1.8	1.6	1.5	1.4	6.9
11	1.2	1.4	1.5	1.7	1.5	8.9
12	1.1	1.4	1.6	1.6	1.5	9.2
14	1.3	1.6	1.5	1.5	1.9	9.7
15	1.0	1.5	1.5	1.5	1.4	9.6
18	1.3	1.7	1.7	1.6	1.3	8.9
19	1.2	1.3	1.1	1.5	1.5	7.8
20	1.2	1.4	1.6	1.4	1.5	6.2
21	1.1	1.0	1.3	1.3	1.2	5.9
23	1.2	1.5	1.6	1.5	1.4	6.0
25	1.0	0.9	1.3	1.1	1.3	8.9
26	1.1	1.1	1.4	1.3	1.2	7.0
27	1.3	1.6	1.5	1.1	0.9	14.6
28	1.0	1.2	1.5	1.1	1.2	10.1
						
**Patient No.**	**Group III (5% ≥ RSD)**					
	**Day 0**	**Day 2**	**Day 14**	**Day 30**	**Day 90**	**RSD, %**
7	1.4	1.5	1.6	1.5	1.6	4.0
8	1.3	1.4	1.5	1.3	1.4	3.7
24	1.4	1.3	1.3	1.3	1.4	3.2

**Table 4:** Some prognostic factors of AMI patients.

*Prognostic Factors*	Group I	Group II	Group III	Group IV
*Number of patients*	3	2	12	10
*Baseline Troponin I*	2323.5 ± 3940.0	3505.1 ± 4340.7	200.6 ± 287.4	3659 ± 8334.3
*Peak Troponin I*	25,000	25,000	21,465 ± 7454.8	24,874.7 ± 434.0
*Peak/Baseline value of Troponin I*	10.8	7.1	107.0	6.8
*Baseline CKMB*	52.6 ± 85.6	49.2 ± 64.1	21.7 ± 44.9	3.2 ± 3.2
*Peak CKMB*	300	248 ± 73.6	209.8 ± 111.6	221.2 ± 90.1
*Peak/Baseline value of CKMB*	5.7	3.4	9.7	69.1
*Extent of CAD*				
*1 vessel*	2 (66.7%)	0 (0.0%)	4 (33.3%)	3 (30.0%)
*2 vessel*	1 (33.3%)	2 (100%)	4 (33.3%)	3 (30.0%)
*3 vessel*	0 (0.0%)	0 (0.0%)	4 (33.3%)	4 (40.0%)

## Data Availability

The authors confirm that the data supporting the findings of this study are available within the article and its supplementary materials.

## Supplementary Materials

The following are available online at https://www.mdpi.com/2076-3921/10/3/434/s1, Figure S1: Pattern type for AGEs in Group 1, Figure S2: Pattern type for AGEs in Group 2, Figure S3: Pattern type for AGEs in Group 3, Figure S4: Pattern type for AGEs in Group 4, Table S1: Spearman correlation analysis of AGE level in serum sample of AMI patients.
