# Supplementary material for: CUPRAC-Reactive Advanced Glycation End Products as Prognostic Markers of Human Acute Myocardial Infarction
Source: Antioxidants (Basel). 2021 Mar 11;10(3):434. doi: 10.3390/antiox10030434 (PMC7999086; doi:10.3390/antiox10030434)
Supplement: Supplementary file 1 [file antioxidants-10-00434-s001.zip › sup/Table S1.docx]

CUPRAC-reactive Advanced Glycation End Products as Prognostic Markers of Human Acute Myocardial Infarction

Govigerel Bayarsaikhan ^1,†^, Delger Bayarsaikhan ^1,†^, Pyung Chun Oh ^2^, Woong Chol Kang ^2,^* and Bonghee Lee ^1,^*

^1^ Center for Genomics and Proteomics, Lee Gil Ya Cancer and Diabetes Institute, School of Medicine, Gachon University, Incheon City, 406-840, Republic of Korea; govigerel.b@n-sage.com (G.B.); delger.b@n-sage.com (D.B.); (B.L.)

^2^ Department of Cardiology, Gil Medical Center, Gachon University of Medicine and Science, Incheon City, 405-760, Republic of Korea; [likemed@gilhospital.com](mailto:likemed@gilhospital.com) (P.C.O.); (W.C.K.)

* Correspondence: [bhlee@gachon.ac.kr](mailto:bhlee@gachon.ac.kr) (B.L.); kangwch@gilhospital.com (W.C.K.); Tel: +82 32 899 6582 (B.L.); +82 32 466 3054 (W.C.K.); Fax: +82 32 899 6519 (B.L.); +82 32 469 1906 (W.C.K.)

† These authors contributed equally to this work.

**
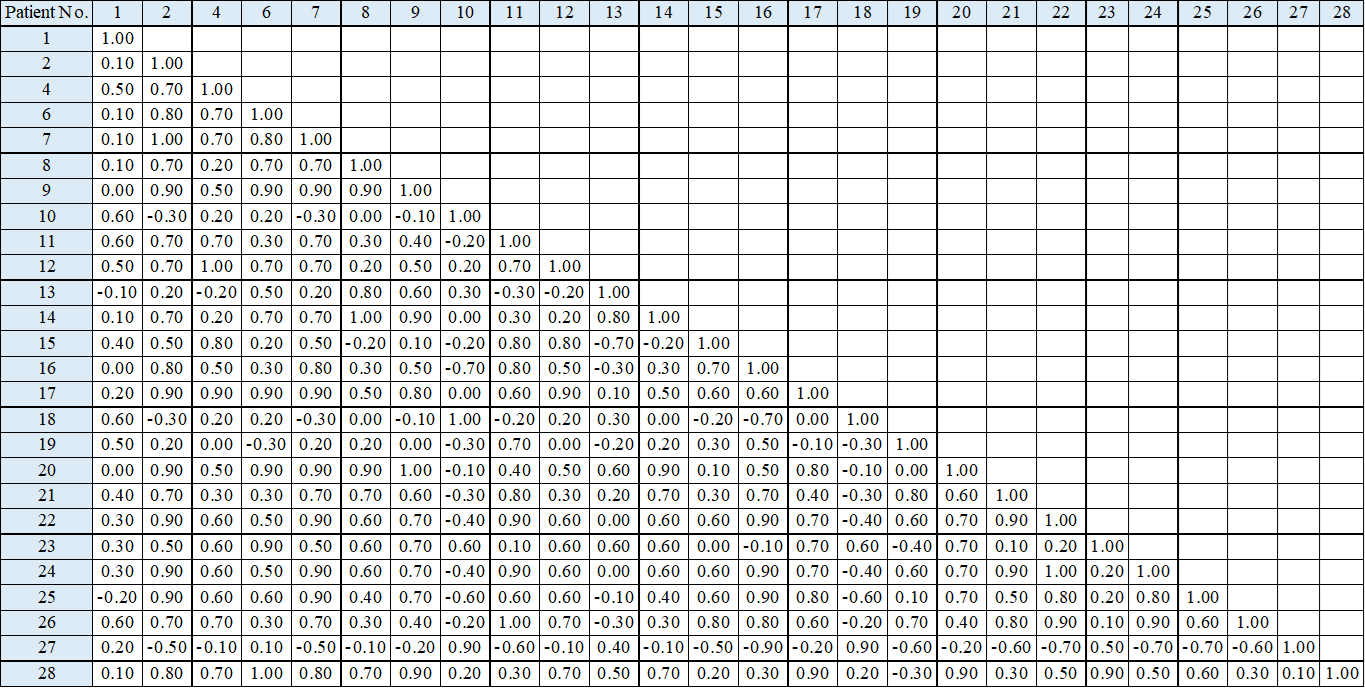
Table S1.** Spearman correlation analysis of AGE level in serum sample of AMI patients
