# Supplementary figures and images for: CUPRAC-Reactive Advanced Glycation End Products as Prognostic Markers of Human Acute Myocardial Infarction
Source: Antioxidants (Basel). 2021 Mar 11;10(3):434. doi: 10.3390/antiox10030434 (PMC7999086; doi:10.3390/antiox10030434)

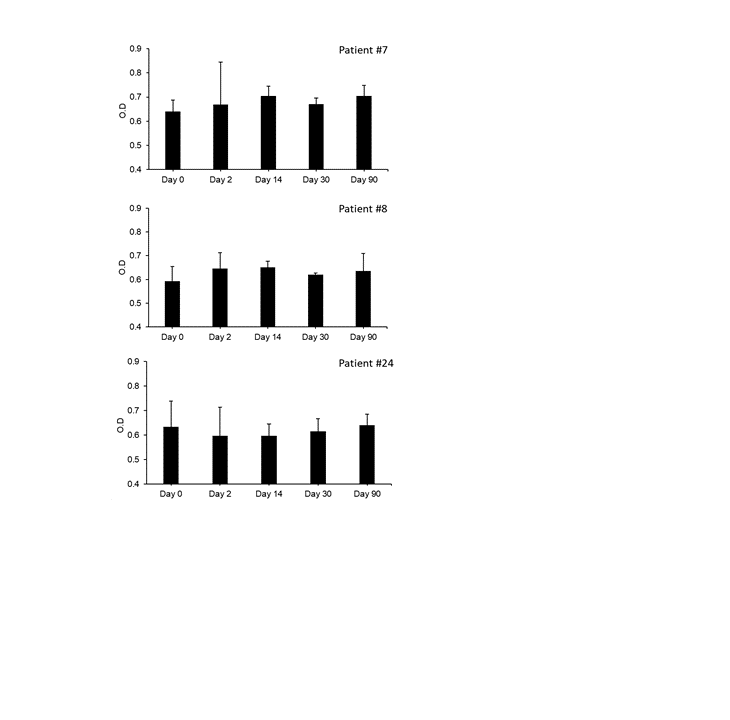

Supplement: Supplementary file 1 [file antioxidants-10-00434-s001.zip › sup/Figure S1.png]

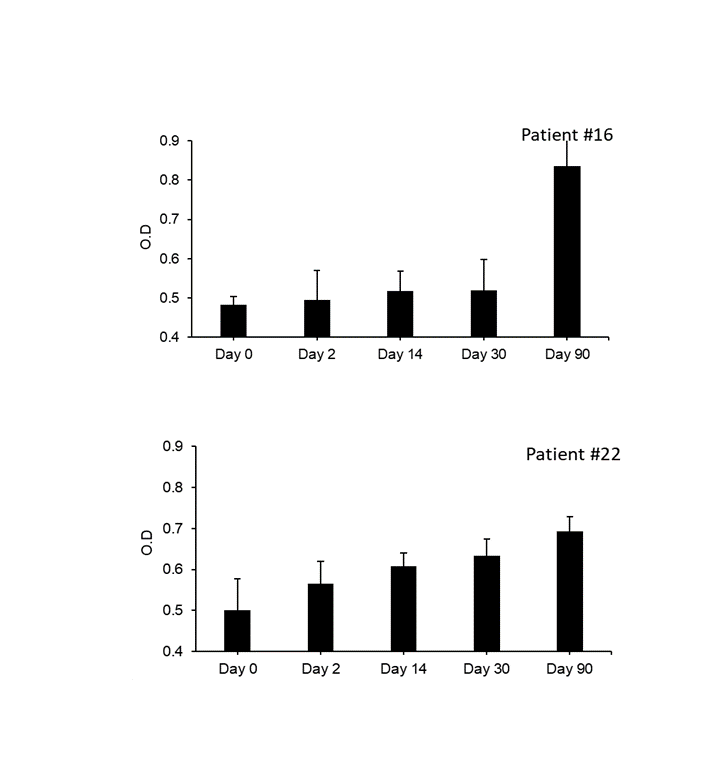

Supplement: Supplementary file 1 [file antioxidants-10-00434-s001.zip › sup/Figure S2.png]

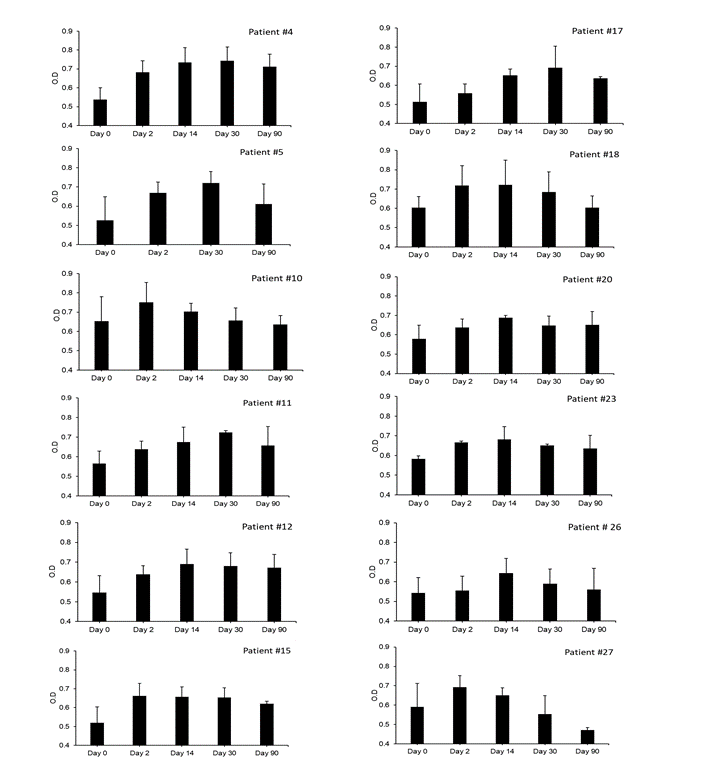

Supplement: Supplementary file 1 [file antioxidants-10-00434-s001.zip › sup/Figure S3.png]

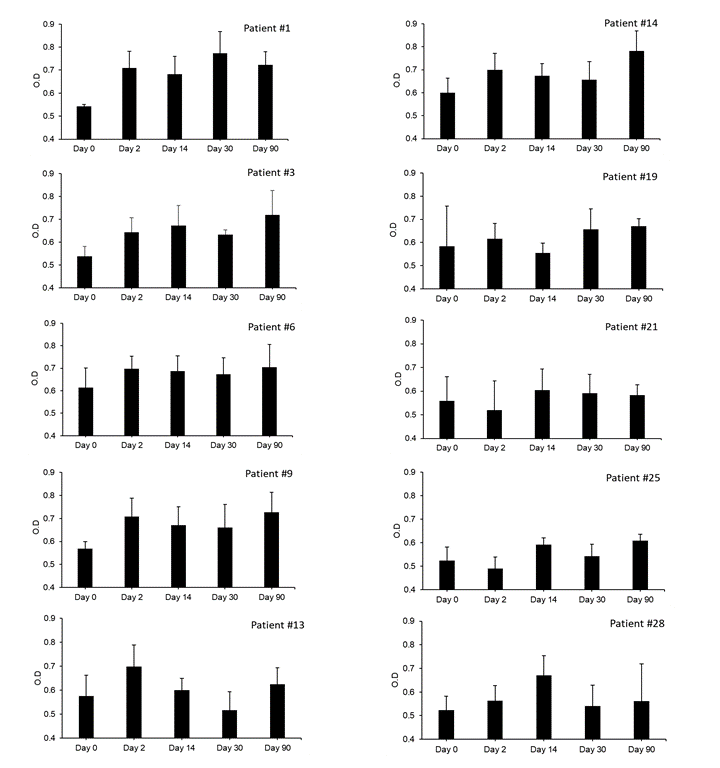

Supplement: Supplementary file 1 [file antioxidants-10-00434-s001.zip › sup/Figure S4.png]
